# CD317 Promotes the survival of cancer cells through apoptosis-inducing factor

**DOI:** 10.1186/s13046-016-0391-2

**Published:** 2016-07-22

**Authors:** Xin Li, Guizhong Zhang, Qian Chen, Yingxue Lin, Junxin Li, Qingguo Ruan, Youhai Chen, Guang Yu, Xiaochun Wan

**Affiliations:** Division of Immunology, School of Fundamental Medicine, Jinzhou Medical University, Jinzhou, 121001 People’s Republic of China; Institute of Biomedicine and Biotechnology, Shenzhen Institutes of Advanced Technology, Chinese Academy of Sciences, Shenzhen, 518055 People’s Republic of China; 713 Stellar-Chance Laboratories, Department of Pathology and Laboratory of Medicine, University of Pennsylvania Perelman School of Medicine, Philadelphia, PA 19104 USA; Shenzhen Institutes of Advanced Technology, Chinese Academy of Science, Shenzhen University Town, 1068 Xueyuan Avenue, Shenzhen, 518055 People’s Republic of China

**Keywords:** CD317, Apoptosis, Apoptosis inducing factor (AIF)

## Abstract

**Background:**

Low nutrient environment is a major obstacle to solid tumor growth. However, many tumors have developed adaptive mechanisms to circumvent the requirement for exogenous growth factors.

**Methods:**

Here we used siRNA interference or plasmid transfection techniques to knockdown or enhance CD317 expression respectively, in mammalian cancer cells, and subjected these CD317-manipulated cells to serum deprivation to study the role of CD317 on stress-induced apoptosis and the underlying mechanism.

**Results:**

We report that CD317, an innate immune gene overexpressed in human cancers, protected cancer cells against serum deprivation-induced apoptosis. In tumor cells, loss of CD317 markedly enhanced their susceptibility to serum deprivation-induced apoptosis with no effect on autophagy or caspase activation, indicating an autophagy- and caspase-independent mechanism of CD317 function. Importantly, CD317 knockdown in serum-deprived tumor cells impaired mitochondria function and subsequently promoted apoptosis-inducing factor (AIF) release and nuclear translocation but had little effect on mitochondrial and cytoplasmic distributions of cytochrome C, a pro-apoptotic factor released from mitochondria that initiates caspase processing in response to death stimuli. Furthermore, overexpression of CD317 in HEK293T cells inhibits serum deprivation-induced apoptosis as well as the release and nuclear accumulation of AIF.

**Conclusion:**

Our data suggest that CD317 functions as an anti-apoptotic factor through the mitochondria-AIF axis in malnourished condition and may serve as a potential drug target for cancer therapy.

**Electronic supplementary material:**

The online version of this article (doi:10.1186/s13046-016-0391-2) contains supplementary material, which is available to authorized users.

## Background

Hypoxia, low nutrient environments, and exogenous growth factor depletion are among the most important challenges that any rapidly growing solid tumor must surmount to survive, grow and metastasize. Serum or growth factor deprivation can induce apoptosis in normal cells as well as tumor cells that cannot mount an adaptive response to it [1–3]. Despite all this, many tumors have developed adaptive mechanisms to circumvent this requirement for exogenous growth factors [1]. These adaptive mechanisms are important for tumor growth and invasion but still remain unclear, which limits the strategy design for cancer therapy.

CD317 (also referred as tetherin, BST2, or HM1.24 antigen) is a type II transmembrane glycoprotein with an atypical membrane topology [4]. It consists of an N-terminal transmembrane domain, extracellular coiled-coiled domain, and a C-terminal GPI anchor [4]. This unique topology gives CD317 viral tethering function [5–8]. As reported, CD317 inhibits the release of many enveloped viruses from the surface of infected cells. These include all retroviruses tested as well as members from seven other families, such as *Hepadnaviridae* (Hepatitis B virus), *Flaviviridae* (Hepatitis C virus), *Filoviridae* (Ebola and Marburg viruses), *Arenaviridae* (Lassa fever virus), *Herpesviridase* (Kaposi’s sarcoma-associated herpesvirus), *Paramyxoviridae* (Sendai virus and Nipah virus), and *Rabdoviridae* (vesicular stomatitis virus) [6, 9–15]. There is a growing literature demonstrating the importance of CD317 in limiting viral infection, however, other functions of CD317 such as its impact on tumorigenesis remain undefined. CD317 expresses in several types of cancers including multiple myeloma (MM), B cell lymphoma, lung cancer, head and neck squamous cell carcinomas, endometrial cancer, brain cancer and bone metastatic breast cancer [9]. Although it is unclear what function CD317 serves on transformed cells, it was found that overexpression in breast cancer cells results in increased migration and proliferation [16]. In addition, CD317 is a potential target for tumor immunotherapy. Humanized monoclonal antibody (McAb) against CD317 showed significant tumor growth inhibition and prolonged survival in human MM xenograft models and MM patients, and the antitumor effect of CD317 McAb were largely mediated by natural killer (NK) cell and monocyte- and macrophage-mediated antibody-dependent cellular cytotoxicity (ADCC) [17]. In our present study, we investigated the anti-apoptotic effect of CD317 on several mammalian cell lines cultured in serum deprivation condition, and explored the underlying mechanisms.

## Methods

### Antibodies and reagents

Antibodies used in this study are as follow: monoclonal rabbit anti-BST-2(Abcam, 1:1000), polyclonal rabbit anti-Bcl-2 (CST, 1:1000), polyclonal rabbit anti-Caspase-3 (CTS, 1:1000), monoclonal mouse anti-Caspase-8(1C12)(CST, 1:1000), polyclonal rabbit anti-Caspase-9 (CST, 1:1000), polyclonal rabbit anti-LC3A/B(CST, 1:1000), polyclonal rabbit anti-AIF (CST, 1:1000), polyclonal rabbit anti-COX IV (CST, 1:1000), monoclonal mouse anti-Lamin A/C(4C11) (CST, 1:1000), monoclonal mouse anti-cytochrome C (Beyotime Biotech, 1:200), polyclonal mouse anti-β-Actin and anti-GAPDH (Santa Cruz Biotechnology, 1:8000), HRP-labeled goat anti-mouse and anti-rabbit IgG (Earthox, 1:10000).

DMEM medium, fetal bovine serum (FBS), penicillin and streptomycin were purchased from HyClone (Logan, USA). L-glutamine was purchased from Gibico (CA, USA). Annexin V-FITC/PI apoptosis detection kit and was purchased from TransGen Biotech (Beijing, China). 7-AAD viability staining solution was purchased from BioLegend (San Diego, CA, USA). Nuclear extraction kit and mitochondria extraction kit were obtained from Pierce Biotechnology (Rockford, USA). Ac-DEVD-CHO was purchased from Beyotime Biotech (Nanjing, China). CD317-specific siRNA (named as siR317) and Normal Control siRNA (name as NC) were synthesized by GenePharma (Shanghai, China). The sequences of the siRNA targeting human CD317 were 5-CCAGGUCUUAAGCGUGAGAdTdT-3 and 5-UCGCGGACAAGAAGUACUAdTdT-3 (corresponding to base pairs 432–450 and 452–470 of the human CD317 sequence, respectively) [18], and the sequences of murine CD317-specific siRNA were 5-GGGUUACCUUAGUCAUCCUdTdT-3 and 5-GCUUGAGAAUGAAGUCACGdTdT-3 (corresponding to base pairs 126–144 and 379–397 of the murine CD317 sequence, respectively), the NC siRNA (5-UUCUCCGAACGUGUCACGUdTdT-3) was used as negative control. MigR1-CD317 plasmid (named as plasCD317) was constructed in our lab. Briefly, the full length of human *CD317* CDS was cloned from Jurkat cells by RT-PCR using specific primers, digested with Bgl II and Xho I, then subcloned into the expression vector MigR1 and sequenced.

### Cells and transfection

Hela (an epithelial cell line from female cervical cancer), SK-OV-3(a human ovarian cancer cell line), MCF-7 (a luminal human breast cancer cell line), HepG2 cells(a hepatocellular carcinoma cell line), sp2/0 cells (a mouse myeloma cell line), U266(a human myeloma cell line) and HEK293T (a CD317 negative human embryonic kidney cell line) were obtained from ATCC or Cell bank, Chinese academy of sciences(Shanghai, China) and routinely culture in DMED medium or RPMI-1640 medium supplemented with 10 % fetal bovine serum, 2 mM L-glutamine, 100 U/mL penicillin, and 10 mg/mL streptomycin. All cultures were maintained in a humidified 5 % CO2 incubator at 37 °C, and routinely passaged when 80–90 % confluent.

The CD317-positive cells were transfected with NC (named as NC cells) or siR317 (named as siR317 cells), while the HEK293T cells were transfected with MigR1 or plasCD317, respectively. All transfections were performed using Lipofectamine 3000 (Invitrogen, USA) or Gene Pulser Xcell (BioRad, USA) according to the manufacturer’s instructions.

After the transfection, cells were cultured in normal or serum deprivation condition for subsequent experiments.

### FACS analysis for apoptosis

After transfection, cells were cultured in normal or serum withdrawal condition for another 48 h and then stained with 7-AAD or double-stained with Annexin V-FITC and propidium iodide (PI) using an apoptosis detection kit for flow cytometry analysis. All samples were analyzed using flow cytometer (FACS Canto II, BD).

### JC-1 assay for MMP

Mitochondrial membrane potential (MMP,ΔΨm) was evaluated by JC-1 reduction according to the manufacturer’s instructions. After indicated treatment and wash, cells were incubated with 2.5 μM JC-1 at 37 °C for 30 min and then quantified using flow cytometer (FACS Canto II, BD).

### Immunofluorescence and microscopy

Hela cells mounted on glass slides were fixed with 4 % paraformaldehyde (PFA) for 20 min at room temperature and then permeabilized with 0.1 % Triton X-100 for another 20 min. Cells were blocked with goat serum for 20 min at room temperature and then incubated with primary antibody (polyclonal rabbit anti-AIF, 1:1000) at 4 °C. After overnight incubation, cells were further stained with rhodamine-conjugated goat anti-rabbit IgG (Molecular Probes), and 4, 6-diamino-2-phenylindole (DAPI, Roche) and analyzed on an optical microscopy (Olympus IX71, Tokyo, Japan). 10–15 high-powered fields were evaluated, and fixed fluorescence images were analyzed by Image-Pro Plus software (Media Cybernetics, Silver Spring, MD).

### Cytoplasmic and nuclear protein extraction

Cell cytoplasm and nucleus protein were collected with NE-PER Nuclear and Cytoplasmic Extraction Reagents (Pierce). In brief, cells were washed with cold PBS and fully suspended in ice-cold CER I by vortex on the highest setting for 15 s, followed by incubation on ice for 10 min. Homogenates were added ice-cold CER II, incubated on ice for 1 min and then centrifuged at 16,000 g for 5 min at 4 °C. The supernatants (cytosol) were collected and the pellets were suspended in ice-cold NER and incubated on ice for 40 min, vortex for 15 s every 10 min. The lysates were again centrifuged at 16,000 g for 5 min, and the supernatants (nucleus) were collected. The distributions of protein in cytosol and nucleus were analyzed by western blot.

### Mitochondria isolation

Isolation of mitochondria was performed using mitochondria isolation kit (Pierce). Briefly, 1 × 10^7^ cells were suspended in 800 μL Reagent A and incubated on ice for exactly 2 min, then were added 10 μL Reagent B followed with another incubation on ice for 5 min. After that, 800 μL Reagent C was added and then the mixture was centrifuged at 700 *g* for 10 min at 4 °C. The supernatant was transferred to a new 2.0 mL tube and centrifuged at 12,000 *g* for 15 min at 4 °C to obtain the mitochondria. At last, the isolated mitochondria were lysed in RIPA buffer containing 1.0 mM PMSF (Beyotime, China) to extract protein for further analysis.

### Immunoprecipitation

Hela cells were cultured in normal or serum-deprivation condition for 24 h, cell lysates were prepared and immunoprecipitated with anti-CD317 or control Ig. The precipitates and lysates were subjected to Western blotting with antibodies for the indicated antigens.

### Western blot

Cells were washed with PBS and lysed with RIPA buffer supplemented with 1.0 mM PMSF. After 15-min incubation on ice, lysates were centrifuged at 12,000 rpm for 20 min at 4 °C. The supernatant was collected, and the protein concentration was measured by bicinchoninic acid (BCA) assay (Beyotime, China). Whole cell lysates (25–50 μg of denatured protein) were separated on a 4–20 % gradient SDS polyacrylamide gel and then transferred to a PVDF membrane (Millipore). The membrane was blocked for 1 h at room temperature in PBST, pH 7.4 (PBS with 0.1 % Tween-20) with 5 % BSA. After washing, the membrane was incubated with primary antibodies overnight at 4 °C, followed by extensive washing and incubation with HRP-conjugated secondary antibodies. At last, protein was visualized by an enhanced chemiluminescense detection kit (Millipore, USA). After stripping, the blot was reprobed with β-Actin or GAPDH antibody (Santa Cruz) to ensure equal loading of total protein.

### Statistical analysis

All data represent at least 3 independent experiments and are expressed as mean ± SEM. Statistical comparisons were made using Student’s *t*-test. *P* < 0.05 was considered statistically significant.

## Results

### CD317 knockdown sensitizes tumor cell to serum deprivation-induced apoptosis

**Fig. 1:**
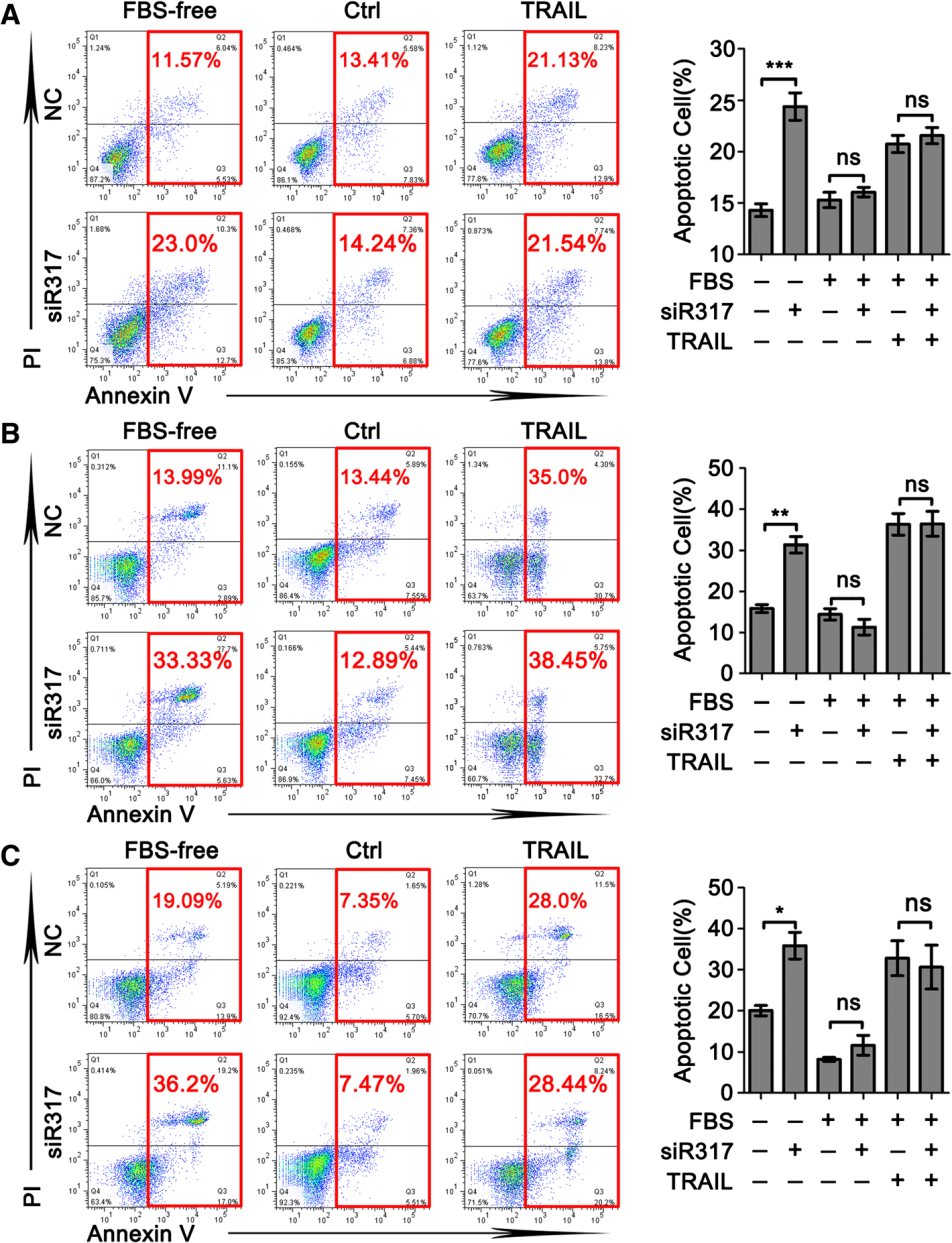
CD317 knockdown enhances serum deprivation-induced apoptosis in tumor cells. (**a**) Representative graphs (*left panel*) and statistical analysis (*Right panel*) of cell apoptosis determined by flow cytometric evaluation in Hela cells. In brief, 36 h post transfection,cells were cultured in the indicated conditions for another 48 h and double stained with annexin V and PI to detect cell apoptosis. ***P < 0.001. (**b**) and (**c**) Representative results obtained from MCF-7 and SK-OV-3, respectively. Cells were treated using the same method described above. *P < 0.05, **P < 0.005

FBS is the most widely used growth supplement in cell culture medium, which provides nutrients and growth factors for cell proliferation. Researchers often use FBS depletion as a means to emulate the tumor microenvironment of some cancers. We, therefore, investigated involvement of CD317 in apoptosis during serum deprivation. As shown in Fig. 1a, CD317 silencing did not affect apoptosis in Hela cells cultured in normal medium (Ctrl group, NC *vs* siR317, 15.29 ± 0.75 % *vs* 16.06 ± 0.46 %, *P* = 0.4036), but markedly enhanced apoptosis during serum deprivation (FBS-free group, NC *vs* siR317, 14.29 ± 0.61 % *vs* 24.40 ± 1.33 %, *P* < 0.0001). Considering that tumor necrosis factor (TNF)-related apoptosis-inducing ligand (TRAIL) is a promising anticancer agent that can induce apoptosis in a wide range of cancers, and the translation of TRAIL into the clinic has been confounded by TRAIL-resistant cancer cell populations [19], we investigated whether CD317 involves in TRAIL-resistant mechanisms. As it is shown in the TRAIL group of Fig. 1a, CD317 did not affect TRAIL-induced apoptosis (NC *vs* siR317, 20.76 ± 0.84 % *vs* 21.57 ± 0.80 %, *P* = 0.4972). In order to further confirm our observation in a broad spectrum of cancer calls, we also examined the other four cell lines in the same experimental condition. We found a consistent effect of CD317 on apoptosis in MCF-7 (Fig. 1b), SK-OV-3 cells (Fig. 1c), HepG2 cells (Additional file 1: Figure S1), U266 cells (Additional file 1: Figure S2) and sp2/0 cells (Additional file 1: Figure S3). These data suggested that CD317 has a function independent of TRIAL and works as an anti-apoptotic factor making the CD317-bearing tumor cells more resistant to serum deprivation-induced apoptosis.

### CD317 knockdown enhances apoptosis through autophagy-independent manner

**Fig. 2:**
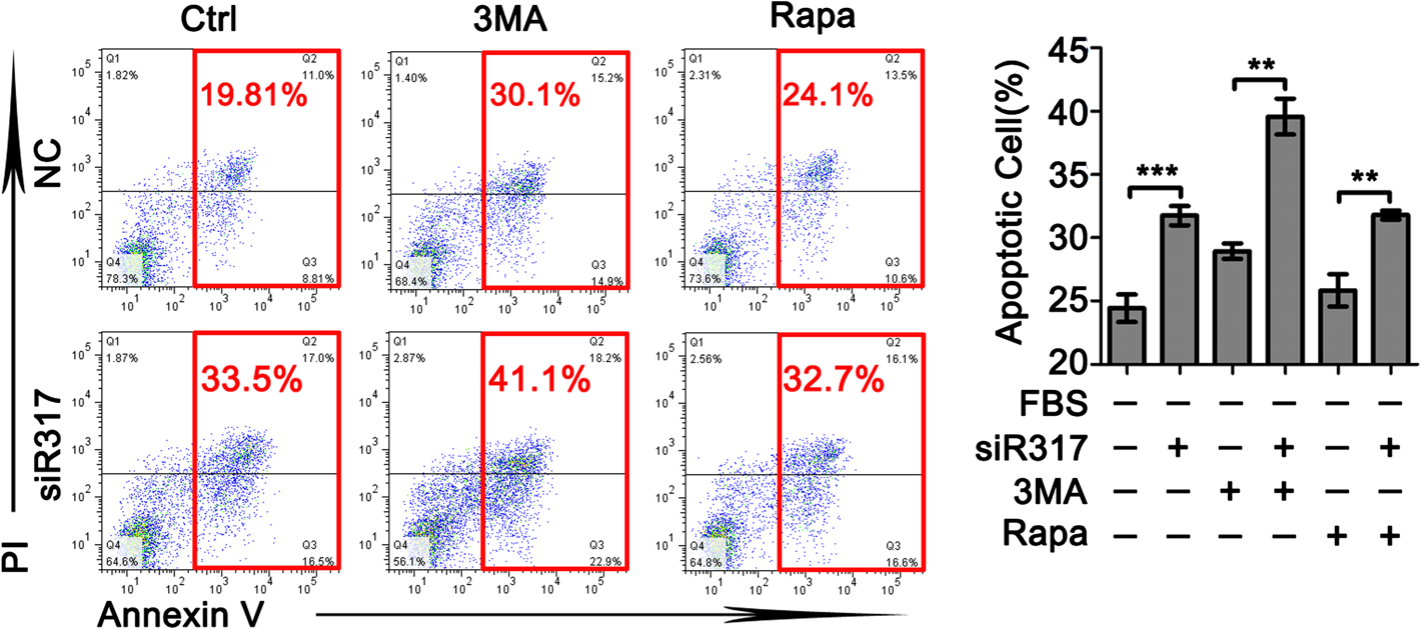
CD317 knockdown enhances apoptosis through autophagy-independent manner. (A) Representative graphs (*left panel*) and statistical analysis (*Right panel*) of cell apoptosis determined by flow cytometric evaluation in Hela cells. Similarly, Cells transfected with NC or siR317 were cultured in the presence or absence of 5 mM 3MA, or 100 nM Rapacycin. After a 48 h-incubation, cells double stained with annexin V and PI to detect cell apoptosis. **P < 0.005, ***P < 0.001

**Fig. 3:**
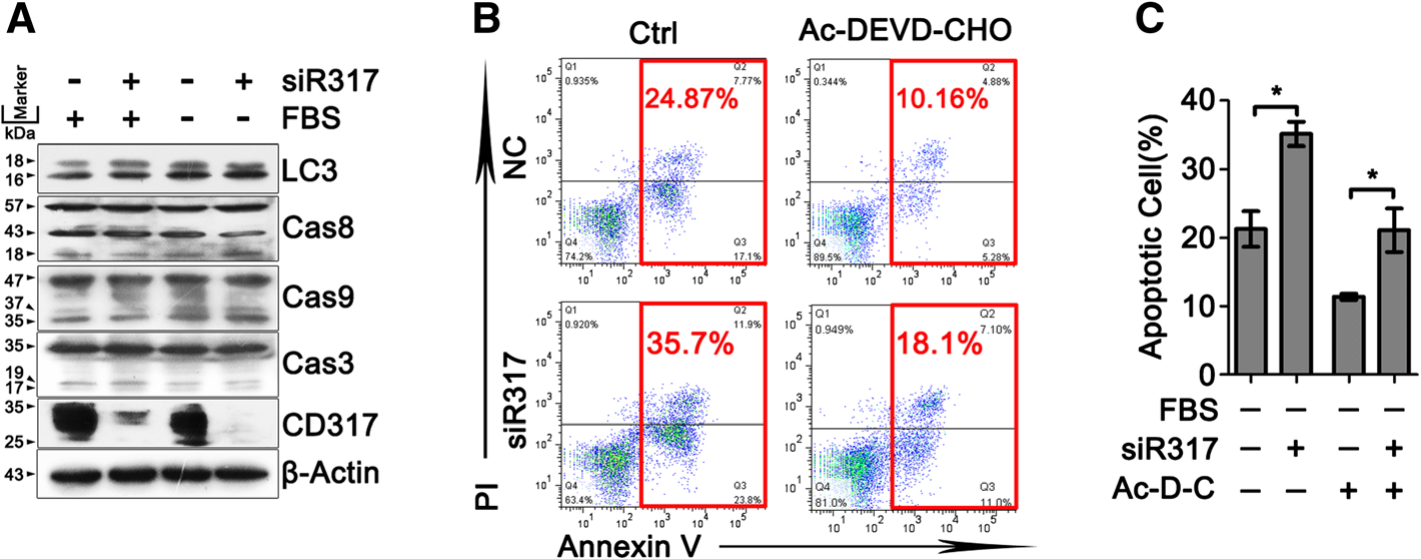
CD317 knockdown promotes cell apoptosis through caspase-independent manner. **a** Blot image represented 1 of 3 independent experiments performed in Hela cells. NC and siR317 cells were cultured in the presence or absence of FBS and harvested at 24 h. Whole cell lysates were prepared and analyzed for LC3, Caspase 8, 9, 3 and CD317. β-actin served as an equal loading control. **b** Representative graphs of cell apoptosis determined by flow cytometric evaluation in Hela cells. NC and siR317 cells were cultured in FBS-free medium with or without 40 μM Ac-DEVD-CHO (a specific inhibitor for Caspase 3) and harvested at 48 h for apoptosis analysis. **c** Quantitative analysis of cell apoptosis as shown in B. *P < 0.05

Starvation induces a vigorous autophagic response to enhance cellular survival, whereas nutrient and serum supplementation inhibit autophagy and induce an intensive transcriptional burst that enables cellular proliferation [20]. To determine whether autophagy flow is responsible for the CD317-mediated apoptosis resistance, we subjected Hela cells to serum withdrawal for 48 h in the presence of either 3MA or rapamycin. As shown in Fig. 2, inhibition of autophagy by 3MA led to an increased apoptosis, while treatment with autophagy agonist (rapamycin) had no significant effect in serum deprivation-induced apoptosis in both NC and siR317 cells. However, neither 3MA nor rapamycin abrogated the anti-apoptotic effect of CD317. In addition, we dynamically monitored autophagosome formation by immunofluorescence in Hela cells cultured in serum-free medium, and found no obvious difference between NC and siR317 group (data not shown). In line with this observation, the level of cleaved LC3, a biochemical marker for autophagic cells, was not affected by CD317 silencing both in normal and serum-free condition (Fig. 3). These results suggested that autophagy flow dose not directly contribute to CD317-mediated apoptosis resistance in serum-deprived tumor cells.

### CD317 knockdown enhances apoptosis through caspase-independent manner

To study whether CD317 affect apoptosis through caspase processing axis, we compared the level of cleaved caspase between NC and siR317 cells. However, no significant difference in the expression of cleaved caspase 8, 9 and 3 was observed between NC and siR317 cells in the presence or absence of FBS, indicating that CD317 silencing cannot influence caspase cascade (Fig. 3a). In accordance with that, treatment with Ac-DEVD-CHO, a specific inhibitor for caspase 3, significantly reduced serum deprivation-induced apoptosis but failed to abrogate the different apoptosis levels between NC and siR317 cells (Fig. 3b, c, Ac-DEVD-CHO group, NC *vs* siR317, 11.37 ± 0.44 % *vs* 21.10 ± 3.18 %, *P* = 0.0386). These data suggested that CD317 prevents cells apoptosis without affecting caspase cascade, which can partially explain the absence of CD317 in the regulation of TRAIL-induced apoptosis, an extrinsic apoptotic pathway mainly depend on caspase activation.

### CD317 Knockdown impairs mitochondria function resulting in AIF release and accumulation in nucleus

**Fig. 4:**
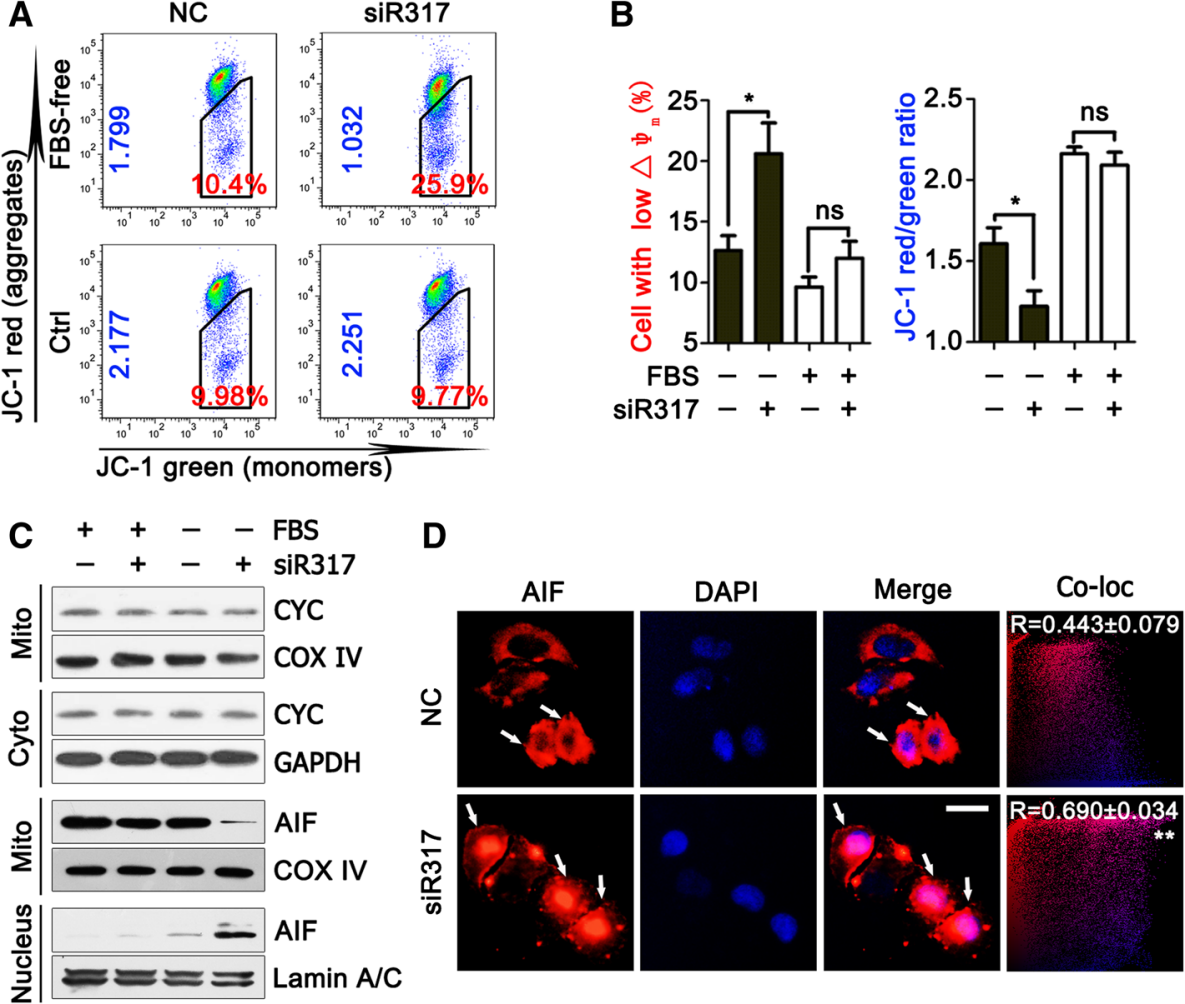
CD317 regulates serum deprivation-induced apoptosis through mitochondria-AIF axis. **a** Flow cytometry in combination with JC-1 staining performed in Hela cells. NC and siR317 cells were cultured in FBS-containing or FBS-free medium. After a 24 h-incubation, cells were harvested and subjected to JC-1 staining following flow cytometry for MMP dection. *Red* characters indicated the percentage of cells with low ΔΨm, while *blue* indicating the JC-1 ratio (*red*/*green* fluorescence). **b** Quantitative analysis of the percentage and JC-1 ratio shown in A. *P < 0.05. **c** Subcellular distribution of cytochrome c (CYC) and AIF in Hela cells detected by western blot. In brief, NC and siR317 cells were cultured in the presence or absence of FBS for 24 h and collected for subcellular fractionation extraction. Lamin A/C, GAPDH, and COX IV served as the loading control for nuclear, cytoplasmic, and mitochondrial protein, respectively. **d** Representative microscopy of immunostained AIF in Hela cells. NC and siR317 cells were harvested after a 24-h incubation with or without FBS, and then fixed and immunostained for AIF (*red*) and nucleus (*blue*). Pictures were taken with a fluorescence microscope. Fifteen microscopic fields per culture were evaluated in a blind manner. Colocalization of AIF and nucleus was analyzed by ImagePro Plus solfware. Pearson’s correlation coefficient (R) was used as a measure of colocalization of *Red* signals with *blue* signals. Correlation plot was corresponding to the left figure. The mean correlation coefficient value (R) ± SEM of at least fifteen fields is shown on the plots. Scale bar: 20 μm

Many studies have shown that cells usually suffered mitochondria dysfunction during serum deprivation, exerting low mitochondrial membrane potential (MMP, ΔΨm) and high permeability, which further caused the release of AIF, a mitochondrial protein that translocated to the nucleus to induce chromatin condensation in a caspase-independent manner [21]. To address whether CD317 suppressed apoptosis of serum-deprived cells through mitochondria-AIF axis, we compared the level of MMP between NC and siR317 cells by JC-1 staining. JC-1 could aggregate in mitochondria and present high red fluorescence in normal cells, whereas exists in the cytoplasm as a monomer emitting green fluorescence in the cells to die. Flow cytometry analysis showed that CD317 silencing had little effect on mitochondrial function in normal condition but markedly promoted serum deprivation-induced MMP loss as observed by a decrease in JC-1 ratio (red/green fluorescence, FBS-free group, NC *vs* siR317, 1.61 ± 0.098 *vs* 1.22 ± 0.097, *P* = 0.0488) as well as an increase percentage of cells with low MMP (FBS-free group, NC *vs* siR317, 12.60 ± 1.23 % *vs* 20.60 ± 2.52 %, *P* = 0.0464) (Fig. 4a–b). These results indicated that CD317 silencing promoted mitochondrial depolarization in serum-deprived tumor cells. Considering that AIF would be released from mitochondria after mitochondrial depolarization and translocate into nucleus to cause cell death, we analyzed the protein distribution of AIF in mitochondria and nucleus. As shown in Fig. 4c, the nuclear level of AIF was significantly higher in serum-deprived siR317 cells than that in the NC counterpart. This nuclear accumulation was accompanied by a decline in mitochondrial distribution, suggesting that CD317 knockdown in serum-deprived cells enhanced AIF release and translocation into nucleus. To address the specificity of this observation, the distribution of cytochrome c in cytoplasm and mitochondria was also detected by western blotting. As shown in Fig. 4c (bottom panel), CD317 knockdown did not cause a visible change in the distribution of cytochrome c in cells cultured in the absence or presences of FBS. Furthermore, microscopy of immunostained AIF demonstrated as well that AIF accumulated more obviously within the nucleus of serum-deprived siR317 cells than that in NC cells (Fig. 4d) as observed by an increase co-location ratio (R value, NC *vs* siR317, 0.44 ± 0.079 *vs* 0.69 ± 0.034, *P* = 0.0062). Thus, these results suggested that CD317 knockdown significantly enhances serum deprivation-induced apoptosis through mitochondria-AIF axis.

To further investigate the resistant mechanism of CD317 in AIF release and nuclear accumulation, we try to find the binding partners of CD317 by immunoprecipitation. As shown in Additional file 1: Figure S4, CD317 did not bind with either AIF or Calpain 1, a Ca^2+^-dependent neutral cysteine protease that colocalizes with AIF and promotes AIF maturation and release [22], in both normal and serum-free condition. These data suggest that CD317 modulates AIF release without direct interaction with AIF or Calpain 1.

### CD317 overexpression inhibits serum deprivation-induces apoptosis in HEK293T cells

**Fig. 5:**
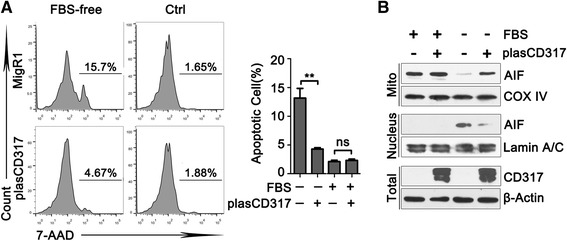
CD317 overexpression inhibits serum deprivation-induced apoptosis in AIF-related manner. **a**. Representative graphs (*left panel*) and statistical analysis (*Right panel*) of cell apoptosis determined by flow cytometric evaluation in HEK293T cells. In brief, 36 h post transfection,cells were cultured in the indicated conditions for another 48 h and stained with 7-AAD to detect cell apoptosis. **P < 0.005. **b**. Subcellular distribution of AIF in HEK293T cells was detected by western blot. In brief, MigR1 and plasCD317 cells were cultured in the presence or absence of FBS for 24 h and collected for total protein or subcellular fractionation extraction. Lamin A/C, β-Actin, and COX IV served as the loading control for nuclear, total, and mitochondrial protein, respectively

To further verify the anti-apoptotic role of CD317, we forced CD317 expression in HEK293T (CD317-negative cells) and detected its influence on apoptosis. Consistent with our previous observations, CD317 overexpression did not affect apoptosis of HEK293T cells cultured in normal medium (Ctrl group, MigR1 vs plasCD317, 2.11 ± 0.24 % vs 2.33 ± 0.23 %, *P* = 0.5403), but markedly suppressed the apoptosis of those cultured with serum deprivation (Fig. 5a, FBS-free group, MigR1 vs plasCD317, 13.17 ± 1.68 % vs 4.28 ± 0.25 %, *P* = 0.0063). In addition, we found that CD317 overexpression significantly inhibited the release and nuclear translocation of AIF in serum-free culture condition (Fig. 5b, further confirmed that CD317 functions as an anti-apoptotic factor through AIF-mediated manner.

## Discussion

In this study, we conducted in vitro experiments to investigate the role of CD317 in five human cell lines and one mouse cell line. Our data demonstrated that CD317 knockdown in either human or mice tumor cells significantly augmented the susceptibility of CD317-bearing tumor cells to serum deprivation-induced apoptosis (Fig. 1, Additional file 1: Figure S1, Additional file 1: Figure S2 and Additional file 1: Figure S3). Moreover, over-expression of CD317 in the CD317-negative cells endowed the cells a markedly increased resistance to the serum deprivation-induced apoptosis (Fig. 5a). These findings revealed a novel function of CD317 in cancer biology and led us to explore the mechanisms underlying this intriguing phenomenon.

CD317 is well documented as a cellular protein which inhibits retrovirus infection by preventing the diffusion of virus particles after budding from infected cells [23]. It has also been identified as the protein that help stabilize lipid rafts by joining nearby lipid rafts to form a cluster, and been predicted to be involved in cell adhesion and cell migration [24]. In the fields of oncology, CD317 has been identified to be overexpressed on several lines of cancer cells [25, 26], and some of its effects on tumors have been reported. In human, overexpression of CD317 has been associated with poor survival of patients with several types cancers [27]. In mice, CD317 expression in breast tumor is associated with tumor size, tumor aggressiveness, and host survival. Knockdown of CD317 in cancer cells decreases metastases to the lung and other distal sites tumor mass [16]. In vitro studies showed that cells with suppressed CD317 have reduced adhesion, anchorage-independent growth, migration, and invasion [16]. Our new finding of the anti-apoptosis function displayed by the CD317-bearing cells in serum-free culture condition could partially reveal the underlying mechanisms of those published observations. Intriguingly, the published report showing that, in two-dimensional culture, mice 4 T1 cells but not E0771 cells with suppressed CD317 expression had higher viability as measured by MTT assay. Therefore, the authors believed that cell viability may not be implicated in the role of CD317 in cancer cell behavior [16]. This is obviously not in line with our observations. The discrepancy may possibly, although not exclusively, be attributable to the differences in experimental design, as it was shown in our data that the effect of CD317 was observed in only the serum-deprived rather than the normal culture condition.

Considering that several groups have discovered that CD317 can activate NF-kB, a transcriptional activator that leads to the rapid expression of proteins involved in cell survival [28], we added TRAIL in the functional assay of CD317. As shown in Fig. 1, TRAIL-induced apoptosis was not affected by CD317-silencing, implying the absence of CD317 in TRIAL-mediated extrinsic apoptotic pathway. To further understand how CD317 endows the cells with the anti-apoptotic function, we tested whether autophagy flow is responsible for the CD317-mediated apoptosis resistance. As shown in Fig. 2, treatment with autophagy agonist had no significant effect on both NC and siR317 cells to serum deprivation-induced apoptosis. Moreover, neither agonist nor inhibitor of autophagy could abrogate the anti-apoptotic effect of CD317, and the level of cleaved LC3 was not affected by CD317 silencing both in normal and serum-deprived condition (Fig. 3). These results suggested that autophagy flow dose not directly contribute to anti-apoptosis function of CD317. Then we turned to examine whether CD317 affect apoptosis through caspase cascade (caspase-8/-3 or caspase-9/-3 axis). We compared the level of cleaved caspases between NC and siR317 cells, and observed no significant difference in the expression of cleaved caspase-8, -9 and -3, indicating that CD317-knowdown has no significant influence on these caspase cascades (Fig. 3a). In accordance with this, treatment with caspase-3 specific inhibitor significantly reduced serum deprivation-induced apoptosis but failed to abrogate the different apoptosis levels between NC and siR317 cells (Fig. 3b, c, Ac-DEVD-CHO group), suggesting that both capase-dependent and -independent pathways are involved in the serum-deprived apoptosis, while the anti-apoptotic function of CD317 may be independent of caspase cascade.

It is known that mitochondria damage results in the release of pro-apoptotic protein such as cytochrome c and AIF, which may trigger caspase-dependent or caspase-independent cell death [29, 30]. To get further insights into the pathway through which CD317 plays its anti-apoptotic role, we examined the pro-apoptotic factor AIF by comparing the level of MMP between the untreated and 317 knowdown cells, and found that CD317-silencing in serum-deprived cells enhanced MMP loss and AIF release. In addition, a translocation of AIF into nucleus was also observed. This nuclear accumulation was accompanied by a decline in mitochondrial distribution, indicating that CD317 knockdown in serum-deprived cells enhanced AIF release and translocation into nucleus. These results suggested that CD317 may protect cells from serum deprivation-induced apoptosis through AIF-mediated caspase-independent cell death pathway.

Interestingly, we observed that CD317 knockdown significantly enhanced AIF release with little effect on the subcellular distribution of cytochrome C (Fig. 4c). To clarify the mechanism underlying this observation, we tried to identify the direct target molecule for CD317. Considering the published evidence showing that mitochondrial calpain 1, a Ca^2+^-dependent neutral cysteine protease, can colocalize with AIF [22], we speculated that AIF or Calpain 1 may be the binding partner of CD317 and tried to confirm this assumption by Co-IP, but as shown in Additional file 1: Figure S4, CD317 did not bind with either AIF or Calpain 1 in both normal and serum-free condition. Further investigations are thus needed to elucidate the bridging molecules between CD317 and AIF.

**Fig. 6:**
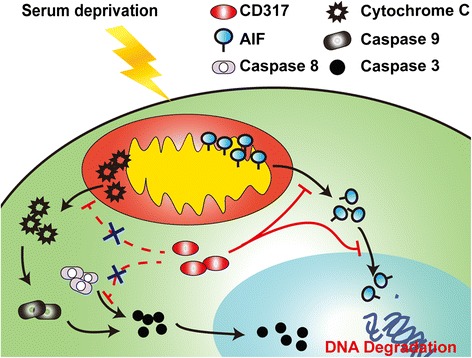
Proposed role of CD317 in apoptosis. In response to death stimulus, mitochondria swelled, dropt mitochondrial membrane potential (MMP) and aggrandized the permeability, resulting in the release of apoptotic molecules such as cytochrome c and AIF. Cytochrome c initiated caspases processing in cytoplasm and ultimately induced cell apoptosis, while AIF translocated to the nucleus to induce chromatin condensation in a caspase-independent manner. CD317 plays an inhibitory role in the mitochondria-AIF axis, protects cells from nutrient deficient-induced apoptosis

Cancer has become a major public health problem over the world due to its increasing incidence and mortality, as well as limited therapy strategies [31, 32]. Over the past two decades, gene therapy, mediated by viral and non-viral vectors, has been developed as a promising pharmacological approach to provide potential treatment options for a variety of cancers [33]. A successful cancer gene therapy requires an appropriate gene which displays selective toxicity toward tumor cells without eliciting harmful effects in normal cells or tissues [34]. CD317 was dramatically upregulated in many tumor cells and involved in tumor growth and metastasis [9, 35]. In this study, we further uncover the anti-apoptotic role and mechanism of CD317 in serum-deprived tumor cells, strongly suggesting that CD317 may be an appropriate target for tumor therapy. Two aspects described as follows can further explain this issue: 1) As we known, tumors may experience malnutrition during the course of their malignant progression, or following the treatments that disrupt tumor vasculature and lead to the stress of prolonged serum growth factor deprivation, and adaptation to such unfavorable conditions is crucial for tumor survival, growth and metastasis [1]. In this study, we confirmed that CD317-silencing increased the susceptibility of tumor calls to apoptosis, and importantly, this effect of CD317-silencing was only observed in serum-deprived cells, implying that gene interference targeting CD317 might be used as a combined approach for the antiangiogenic tumor therapies; 2) One of the major challenges in cancer therapy is that many tumor cells carry mutations in key apoptotic genes such as *p53*, *bcl* family proteins or those affecting caspase signaling, thus making traditional chemotherapeutic agents ineffective. The triggering of caspase-independent death pathways, therefore, has become an attractive alternative approach to eradicating tumor cells [30]. The results of this study as summarized in our model (Fig. 6.) reveal the role of CD317 in protecting tumor cells from nutrient deficient-induced apoptosis and the mechanism that CD317 plays an inhibitory role in the mitochondria-AIF axis, suggesting that CD317 silencing could be a considerable approach for combined cancer therapies. Recently, Zheng Hu et al reported low-intensity ultrasound combined with 5-FU produced much enhanced synergistic anti-tumor effects via enhanced mitochondria-cytochrome c-caspase cascades [36]. However, the anti-tumor effects could only be partially rescued by pan-caspase inhibitor, strongly suggesting that another mitochondrial pro-apoptotic factor such as AIF may be involved in this process. Thus, CD317 interference may further enhance US + 5-FU treatment-mediated anti-tumor effects through promoting AIF release and nuclear accumulation.

## Conclusion

Taken together, our data for the first time revealed that under a nutrient deficient condition, CD317 functions as an anti-apoptotic factor through AIF-mediated caspase- and autophagy-independent manner and may serve as a target for the development of new therapeutics for CD317-positive cancers.

## Abbreviations

3MA, 3-methyladenine; 5-FU, 5-fluorouracil; 7-AAD, 7-aminoactinomycin D; AIF, apoptosis-inducing factor; BCA, bicinchoninic acid; BSA, bovine serum albumin; BST-2, bone marrow stromal cell antigen 2; CDS, coding sequence; Co-IP, Co-Immunoprecipitation; DAPI, 4,6-diamino-2-phenylindole; EDTA, ethylene diamine tetraacetic acid; FBS, fatal bovine serum; FITC, fluorescein isothiocyanate; McAb, monoclonal antibody; MMP, mitochondrial membrane potential; NC, normal control; PBS, phosphate buffer saline; PFA, paraformaldehyde; PI, propidiumiodide; PMSF, phenylmethylsulfonyl fluoride; PVDF, polyvinylidene fluoride; RIPA, radio-immunoprecipitation assay; SDS, sodium dodecyl sulfate; SEM, standard error of mean; US, ultrasound

## Additional file

Additional file 1: Supporting Online Material for “CD317 Promotes the survival of cancer cells through apoptosis-inducing factor”. (DOCX 550 kb)
