# Supplementary material for: CD317 Promotes the survival of cancer cells through apoptosis-inducing factor
Source: J Exp Clin Cancer Res. 2016 Jul 22;35:117. doi: 10.1186/s13046-016-0391-2 (PMC4957287; doi:10.1186/s13046-016-0391-2)
Supplement: Additional file 1: — Supporting Online Material for “CD317 Promotes the survival of cancer cells through apoptosis-inducing factor”. (DOCX 550 kb) [file 13046_2016_391_MOESM1_ESM.docx]

**Supplemental figure and figure legend**


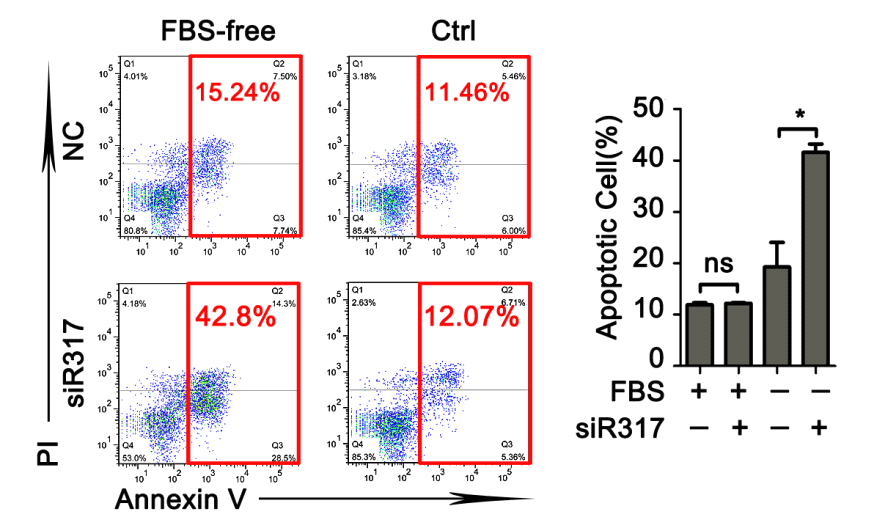


***Figure S1. CD317 knockdown enhances serum deprivation-induced apoptosis in HepG2 cells.*** Representative graphs (left panel) and statistical analysis (Right panel) of cell apoptosis determined by flow cytometric evaluation in HepG2 cells. In brief, 36 h post transfection，cells were cultured in the indicated conditions for another 48 h and double stained with annexin V and PI to detect cell apoptosis. **P*<0.05.


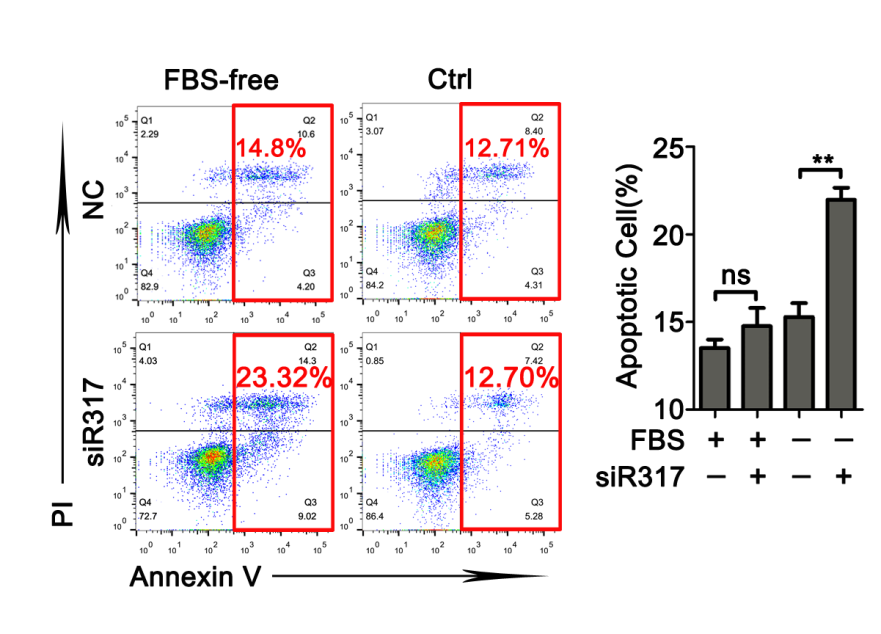


***Figure S2.*** ***CD317 knockdown enhances serum deprivation-induced apoptosis in U266 cells.*** Representative graphs (left panel) and statistical analysis (Right panel) of cell apoptosis determined by flow cytometric evaluation in sp2/0 cells. ***P*<0.01.


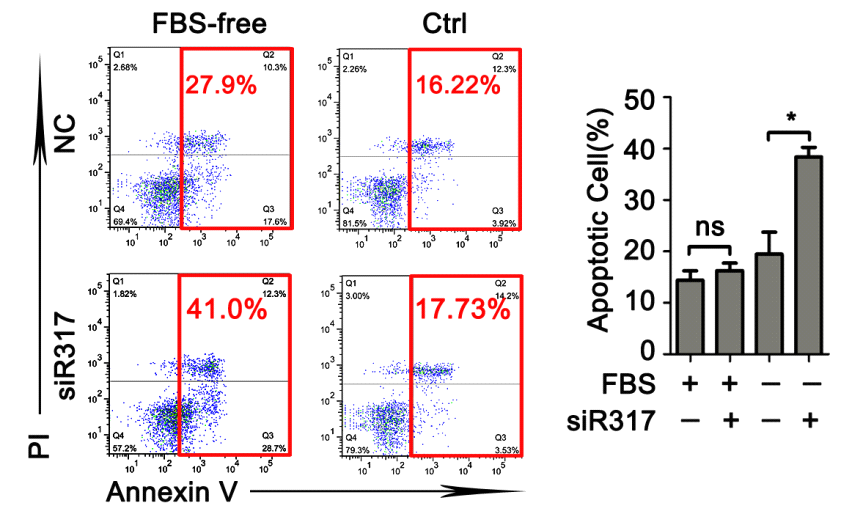


***Figure S3.*** ***CD317 knockdown enhances serum deprivation-induced apoptosis in sp2/0 cells.*** Representative graphs (left panel) and statistical analysis (Right panel) of cell apoptosis determined by flow cytometric evaluation in sp2/0 cells. **P*<0.05.


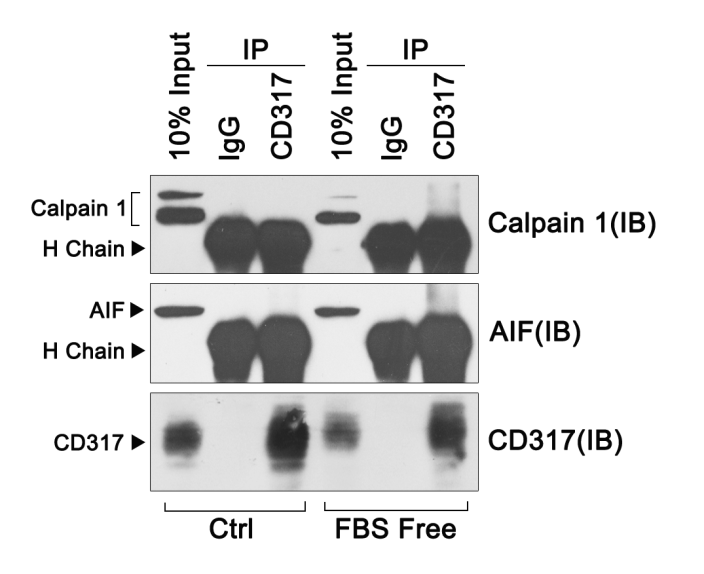


***Figure S4. CD317 cannot bind with either AIF or Calpain 1.*** Hela cells were cultured in the indicated conditions. 24 h later, cell lysates were prepared and immunoprecipitated with anti-CD317 or control Ig. The precipitates and lysates were subjected to Western blotting with antibodies for the indicated antigens.
